# Explore Protein Conformational Space With Variational Autoencoder

**DOI:** 10.3389/fmolb.2021.781635

**Published:** 2021-11-12

**Authors:** Hao Tian, Xi Jiang, Francesco Trozzi, Sian Xiao, Eric C. Larson, Peng Tao

**Affiliations:** ^1^ Center for Research Computing, Center for Drug Discovery, Design, and Delivery (CD4), Department of Chemistry, Southern Methodist University, Dallas, TX, United States; ^2^ Department of Statistical Science, Southern Methodist University, Dallas, TX, United States; ^3^ Department of Computer Science, Southern Methodist University, Dallas, TX, United States

**Keywords:** protein system, conformational space, variational autoencoder, molecular dynamics, deep learning

## Abstract

Molecular dynamics (MD) simulations have been actively used in the study of protein structure and function. However, extensive sampling in the protein conformational space requires large computational resources and takes a prohibitive amount of time. In this study, we demonstrated that variational autoencoders (VAEs), a type of deep learning model, can be employed to explore the conformational space of a protein through MD simulations. VAEs are shown to be superior to autoencoders (AEs) through a benchmark study, with low deviation between the training and decoded conformations. Moreover, we show that the learned latent space in the VAE can be used to generate unsampled protein conformations. Additional simulations starting from these generated conformations accelerated the sampling process and explored hidden spaces in the conformational landscape.

## 1 Introduction

Molecular dynamics (MD) simulations have been applied extensively to understand protein structure, function, and kinetics. Klepeis et al. (2009); Song et al. (2020) Through the development of hardware and software, e.g., graphics processing unit (GPU) Shaw et al. (2009) and OpenMM Eastman et al. (2017), the simulation time scale has climbed from nanoseconds to milliseconds. However, this time scale is still insufficient in the study of slow-motion molecular events such as large-scale conformational transitions. Hartmann et al. (2014) Moreover, the energy landscapes of proteins are discretized with many local energy minima separated by high energy barriers. Krivov (2011) This rough energy landscape limits the applications of MD simulations and hinders a complete sampling of protein movements.

In recent years, enhanced sampling methods have been developed to address this issue. One class of methods introduces biasing potentials, such as Gaussian-accelerated MD (GaMD) Hamelberg et al. (2004), to expand the landscape. However, some domain knowledge is required to define the essential coordinates, e.g., collective variables (CVs). Maximova et al. (2016) Another class iteratively conducts new simulations by selecting seed structures from less sampled regions. Those starting structures can be chosen from the results of Markov state models Bowman et al. (2010) or dimensionality reduction methods.

The advancement of deep learning provides an alternative approach for protein sampling. Several studies have demonstrated the success of both autoencoders (AEs) and variational autoencoders (VAEs) in their applications to protein conformations and functions (Degiacomi, 2019; Lemke and Peter, 2019; Tsuchiya et al., 2019; Jin et al., 2021; Ramaswamy et al., 2021). These models are capable of learning a low-dimensional representation through the encoder model while predicting new protein conformations through the decoder model. Moreover, the learned latent space in one protein system is biologically meaningful and can be transferred to a similar system, with latent variables be treated as CVs. (Sultan et al., 2018).

In this study, we proved the success of variational autoencoders in protein sampling by using the enzyme adenosine kinase (ADK) as an example. The crystallized ADK is initially in its closed state and undergoes a series of conformational changes to its open state (Schrank et al., 2013). MD simulations were conducted to sample this process and used for model training. A benchmark study was conducted to compare the performance of both VAEs and AEs with regard to the encoder and decoder models. VAEs perform better than AEs and are selected for further analysis. Random points in the middle of the closed and open states in the latent space were selected and decoded into new protein conformations. Additional MD simulations starting from these predicted conformations, together with the training simulations, sampled a complete transition from the closed to the open states and explored hidden conformational spaces.

## 2 Methods

### 2.1 Molecular Dynamics Simulations

The initial structures of the closed and open states of ADK were taken from the Protein Data Bank (PDB) Berman et al. (2000) with the PDB IDs as 1ake and 4ake, respectively. Chain A was extracted with the removal of ligands and crystal waters as the starting structure in both states. The systems were added with hydrogen atoms and solvated in a periodic boundary box of TIP3P water molecules Jorgensen et al. (1983). Na^+^ and Cl^−^ ions were used to neutralize the system. Energy minimization was performed with the steep descent method for each system. 100 picoseconds of canonical ensemble (NVT) Langevin MD simulations were carried out, followed by 200 picoseconds of isothermal–isobaric ensemble (NPT) simulations at 1 atm and 300 K. Finally, the systems were switched back to NVT. Both the closed and open states conducted 5 nanoseconds simulations initially while the closed state simulations continued to 50 nanoseconds. Each MD simulation was repeated three times independently. The electrostatic interactions were calculated using the particle mesh Ewald (PME) algorithm (Essmann et al., 1995). Bonds associated with hydrogen atoms were constrained using the SHAKE algorithm Ryckaert et al. (1977) with 2 fs step size. All simulations were conducted with CHARMM27 force field (Foloppe and MacKerell, 2000) and OpenMM 7 Eastman et al. (2017).

Trajectories were aligned to the first frame and 1,660 heavy backbone atoms were selected. The Cartesian coordinates were extracted and further normalized as features using the MinMax scaling. Coordinate *c*, (*c* ∈ *x*, *y*, *z*) for atom *i* in structure *k* is normalized as:

$$\text{normed }c_{i}^{k}=\frac{c_{i}^{k}-\min\left(c\right)}{\max\left(c\right)-\min\left(c\right)}$$ (1)

### 2.2 Autoencoders and Variational Autoencoders

**FIGURE 1:**
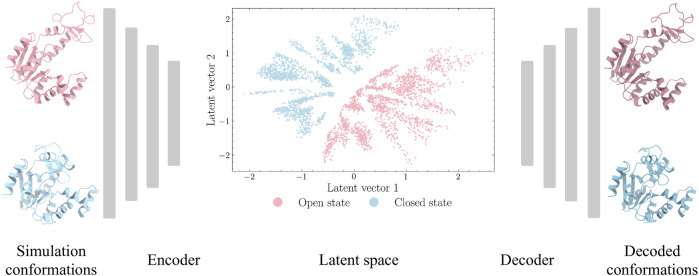
Autoencoder architecture for ADK protein. The Cartesian coordinates from the closed and open states of ADK trajectories are extracted as inputs. The encoder module is designed with decreasing number of neurons in hidden layers to encode high-dimensional inputs to a low-dimensional latent space. The decoder module, with increasing number of neurons in hidden layers, aims to project latent space back to protein structures.

Autoencoders are a type of unsupervised deep learning models that are designed to encode an input to a low-dimensional latent space and decode it back (Baldi, 2012). For this purpose, autoencoders normally have a hourglass shaped architecture, as shown in Figure 1. The first part of the hourglass is an encoder module for compression and the later part is a decoder module for reconstruction. The latent vectors are expected to capture the key representational information of the input space.

However, such classical autoencoders fail to learn a useful or well-constructed latent spaces and thus lead to unsatisfactory results in some applications (Wetzel, 2017; Strub and Mary, 2015). These shortcomings limit the application of AEs for a wider range of problems. To address this, variational autoencoders are built upon autoencoders with an additional optimization constraint that latent space follows a certain distribution (like a normal distribution) (Doersch, 2016). Through this constraint, information is evenly distributed in the latent space that enables the model to sample any point for data reconstruction.

The encoder module, an inference model *q*_*ϕ*_(*z*|*x*), and the decoder module, a generative model *p*_*θ*_(*x*|*z*) are simultaneously trained with data *x* and the latent variable *z*. Parameters *ϕ* and *θ* parameterize the encoder and decoder, respectively. VAEs model the joint distribution of the latent space and data as *p*(*x*, *z*) = *p*_*θ*_(*x*|*z*)*p*(*z*). The term *p*(*z*) is a prior over the latent variables which is typically chosen as a normal distribution for ease of sampling. The intractable posterior *p*_*θ*_(*z*|*x*) = *p*_*θ*_(*x*|*z*)*p*(*z*)/(*∫p*_*θ*_(*x*|*z*)*p*(*z*)*dz*) is approximated using the tractable variational Bayes approach which maximizes the Evidence Lower Bound (ELBO):

$$L\left(\phi ,\theta ;x\right)=E_{q_{\phi }\left(z|x\right)}\left[\log p_{\theta }\left(x|z\right)\right]-KL\left(q_{\phi }\left(z|x\right)\|p\left(z\right)\right)\leq \log p_{\theta }\left(x\right)$$ (2)

where *KL* is the Kullback-Leibler divergence.

In our implementation, the autoencoders and variational autoencoders were developed in Python 3.7 using the Keras package with Tensorflow Abadi et al. (2016) backend v2.4.1.

### 2.3 Performance Assessment Criteria

Several previous studies (Alam et al., 2020; Alam and Shehu, 2020; Guo et al., 2020) focused on the evaluation of autoencoders on the generation of nonlinear featurization and the learned nonlinear representations of protein tertiary structures. In the current study, a similar strategy was employed to quantify and compare the performance of autoencoders and variational autoencoders. Specifically, four metrics were chosen as: 1) Spearman correlation coefficient, 2) Pearson correlation coefficient (PCC), 3) root-mean-square deviation (RMSD), and 4) discrete optimized protein energy (DOPE):

1) *Spearman correlation coefficient.* Spearman correlation coefficient is used to quantitatively analyze how well distances between all pairs of points in the original spaces have been preserved in the reduced dimensions. Spearman correlation coefficient is calculated as:

$$\rho =1-\frac{6\sum \overset{2}{\underset{i}{d}}}{n\left(n^{2}-1\right)}$$ (3)

where *d*_*i*_ and *n* are the difference in paired ranks and number of samples, respectively.

2) *Pearson correlation coefficient (PCC).* PCC uses L2 distance (also referred as Euclidean distance) to estimate the linear relation between distances in the original space and the reduced space. PCC is calculated as:

$$r_{xy}=\frac{\sum_{i=1}^{n}\left(x_{i}-\bar{x}\right)\left(y_{i}-\bar{y}\right)}{\sqrt{\sum_{i=1}^{n}\left(x_{i}-\bar{x}\right)}\sqrt{\sum_{i=1}^{n}\left(y_{i}-\bar{y}\right)}}$$ (4)

where *n* is the sample size, $\bar{x},\bar{y}$ are the mean value of distances, respectively. In the current study, *x* and *y* are used to represent distances input feature space and latent space, respectively.

3) *Root-mean-square deviation (RMSD).* RMSD is used to quantify the conformational differences between training and decoded protein conformations. Given a molecular structure *r* with a reference *r* ^0^, RMSD is calculated as:

$$\text{RMSD}=\sqrt{\frac{\sum_{i=1}^{N}{\left(r_{i}^{0}-Ur_{i}\right)}^{2}}{N}}$$ (5)

where *r* and *r* ^0^ are coordinates and normally represented in the Cartesian space. *N* is the number of atoms. U is the transformation matrix for the best-fit alignment between a given structure and its reference structure.

4) *Discrete optimized protein energy (DOPE)*. The DOPE score (Shen and Sali, 2006) has been extensively used in the assessment of both experimentally and computationally generated models (Deka et al., 2015; Khare et al., 2019). The lower the DOPE score, the better the model. DOPE scores were calculated using modeling package MODELLER (Eswar et al., 2006) version 10.1.

The correlation-based metrics have been widely applied in the comparison between dimensionality reduction methods for biomolecules (Tian and Tao, 2020; Trozzi et al., 2021). They are used here for the encoder module to measure how well the information is preserved in the latent space. The remaining metrics, RMSD, and DOPE, are used for the decoder module to compare the differences between the training and decoded structures.

Moreover, to evaluate the quality of deep learning models, two distance-based metrics, maximum mean discrepancy and earth mover’s distance, were applied to compare the training and generated distributions. Following the strategy from a previous study (Alam and Shehu, 2020), RMSDs were calculated as a proxy variable representing the protein tertiary structures.

1) *Maximum mean discrepancy (MMD).* MMD is a statistical analysis to represent distances between projected distributions using mean embeddings of features. MMD is defined by a feature map φ:X→H where H is a reproducing kernel Hilbert space. MMD is calculated as:

$$\text{MMD}\left(P,Q\right)=\|E_{X∼P}\left[\varphi \left(X\right)\right]-E_{Y∼Q}\left[\varphi \left(Y\right)\right]{\|}_{H}$$ (6)

In the current study, MMD is used for the purpose of model comparison and selection. A good model is expected to generate distributions similar to the training sets, leading to small MMD values.

2) *Earth mover’s distance (EMD).* EMD is a measurement to evaluate dissimilarity between two multi-dimensional probability distributions. It is also known as the Wasserstein metric in mathemathics. Analogically, two distributions on a two-dimensional surface could be considered as two piles of a certain amount of earth (dirt). EMD is the least amount of work needed of transforming one pile into the other. EMD is calculated using SciPy v1.5.2 (Virtanen et al., 2020).

Implied time scales were estimated through the construction of Markov state models (MSMs). Based on the coordinates of the protein NMP-LID angle plots, 100 cluster centers were chosen using *k*-means clustering method. Different lag times were set to calculate the transition matrix. The relaxation timescales were estimated using the corresponding second eigenvalue:

$$t\left(\tau \right)=-\frac{\tau }{\ln\lambda_{1}}$$ (7)

where *λ*_1_ is the second eigenvalue and *τ* is the lag time. MSMs and implied timescales were calculated using the PyEMMA package (Scherer et al., 2015) version 2.5.7.

## 3 Results

**FIGURE 2:**
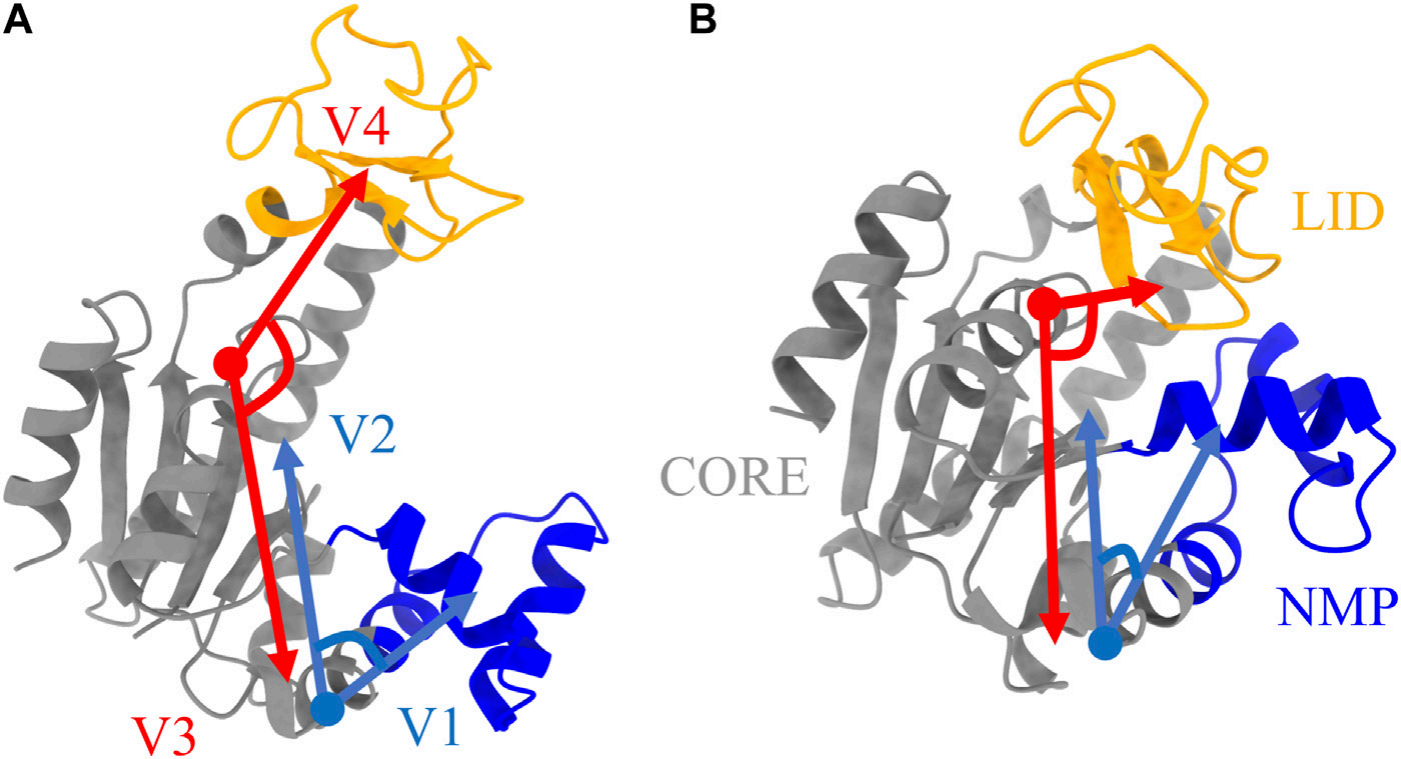
Protein structures of ADK in **(A)** open state and **(B)** closed state. The LID, NMP, and CORE regions are colored as orange, blue, and grey, respectively. Four vectors, V1-V2 for CORE-NMP angle and V3-V4 for CORE-LID angle, are used to characterize protein conformations. The related residues are illustrated in Table 1.

The enzyme adenosine kinase carries out large conformational transitions between the open and closed states in the adenosine triphosphate (ATP) to adenosine diphosphate (ADP) catalysis reaction. Among various structures of ADK, *E. Coli* ADK (abbreviated as ADK) was selected for this study, which is made up of a CORE domain, a LID domain and a NMP domain. According to the previous research (Kubitzki and de Groot, 2008), the CORE domain is relatively rigid while the other two domains are flexible and are known to switch between open and closed conformations. To better characterize the protein conformation, the CORE-LID and CORE-NMP angles were calculated using four vectors. The protein structure, domains and vectors as illustrated in Figure 2.

**FIGURE 3:**
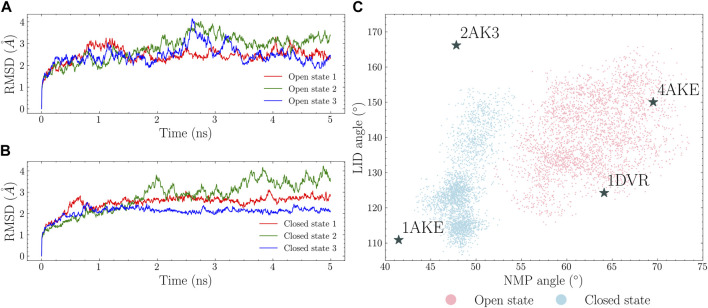
MD simulations of the open and closed states. RMSDs in each trajectory are calculated with regard to the first simulation frame. The open and closed states RMSDs are plotted in **(A)** and **(B)**. NMP and LID angles were calculated and shown in **(C)** with the closed state conformations shown in cyan and the open state conformations in pink.

There are four available crystal structures for ADK: a fully closed state (PDB id: 1AKE), a fully open state (PDB id: 4AKE), a LID-open state (PDB id: 2AK3) and a NMP-open state (PDB id: 1DVR). The fully closed and open states were used for simulations while the other two were used as references. 5 ns MD simulations were conducted for both the open and closed states. The RMSDs were plotted in Figures 3A,B. The four characterizing vectors were also calculated and plotted as a 2D angle map in Figure 3C. Each point in this angle map corresponds to a protein conformation. It is shown that: 1) the open state simulations explored larger conformational spaces compared to the closed state ones; 2) the opening of the LID and NMP domains in the closed state is observed within short simulation time. These suggest that the transition occurs in a short time scale, which aligns with the past findings (Arora and Brooks, 2007; Hanson et al., 2007; Formoso et al., 2015). However, given the limited simulation time, a complete transition path connecting the closed and the open state was not observed. Moreover, there is almost no overlap between the conformational spaces covered by these two states.

The Cartesian coordinates in these 5 ns simulations were scaled and used as the data set for model training. Simulations with an interval of 4 (e.g., 4, 8, …) were extracted as the testing set and the remaining intervals are used as the training set. Therefore, the overall data set was split into 75% for training and 25% for testing. Autoencoders and variational autoencoders with different number of hidden layers were trained using this data set. Detailed model architectures are listed in Table 2.

**TABLE 1:** Residue numbers in the centers of mass of heads and tails in the four vectors as shown in Figure 2.

	1AKE-4AKE		1DVR		2AK3	
	Tail	Head	Tail	Head	Tail	Head
V1	90–99	35–55	95–101, 106–108	39–59	95–104	41–61
V2	90–99	115–125	95–101, 106–108	124–134	95–104	119–129
V3	115–125	179–185	124–134	188–194	119–129	183–189
V4	115–125	125–153	124–134	134–162	119–129	129–157

**TABLE 2:** Architectures of autoencoders and variational autoencoders. The number of neurons in the input and output layers is a fixed number of 4980 while the number of neurons in the encoder and decoder varies with the number of hidden layers. The dimension of the latent space is set to 2.

Input	Hidden layers	Encoder size	Latent space size	Decoder size	Output
4980	1	128	2	128	4980
	2	512, 32		32, 512	
	3	1024, 128, 16		16, 128, 1024	
	4	1024, 256, 64, 16		16, 64, 256, 1024	
	5	2048, 512, 128, 32, 8		8, 32, 128, 512, 2048	

**FIGURE 4:**
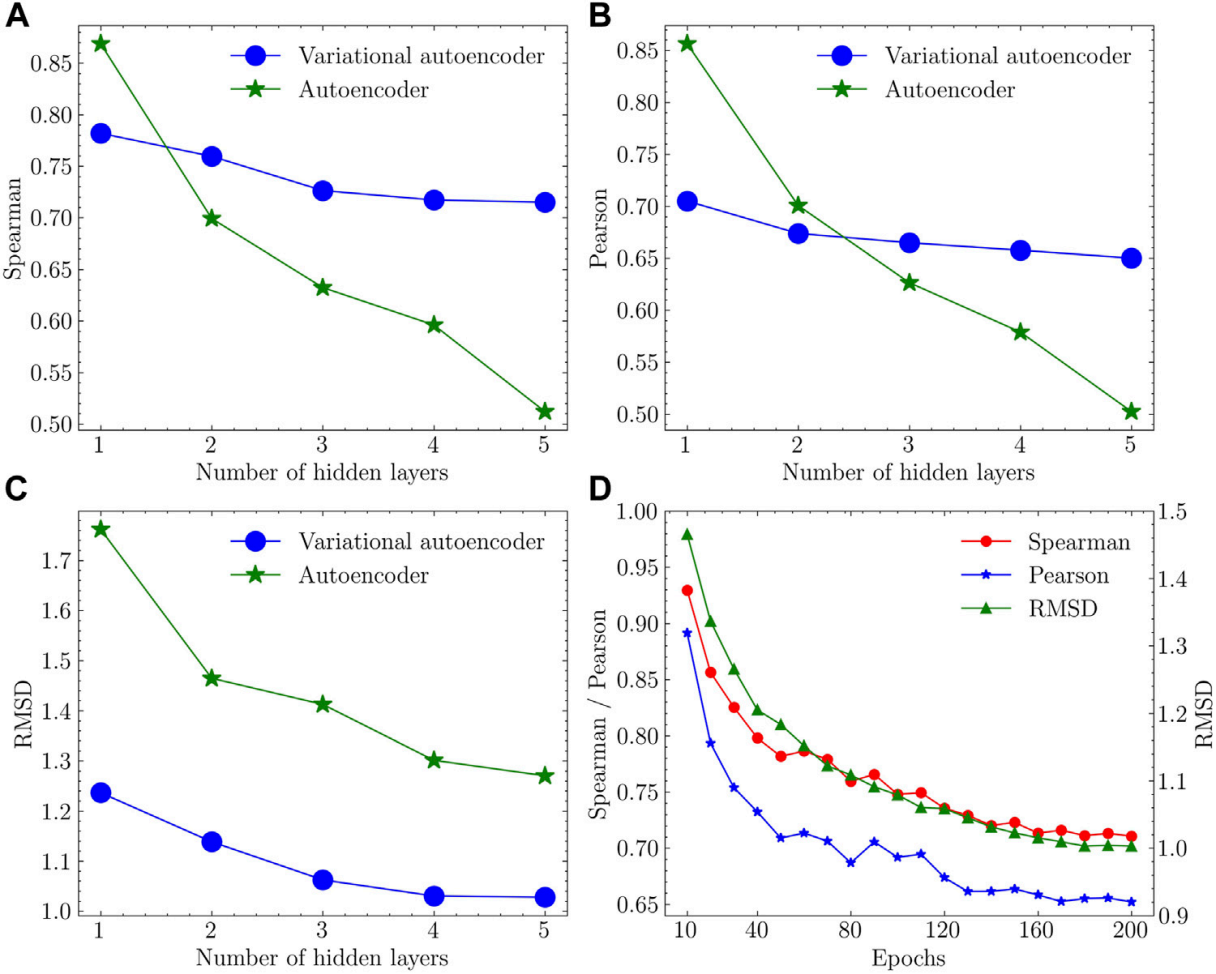
Performance assessment results in **(A)** Spearman correlation coefficient, **(B)** Pearson correlation coefficient and **(C)** RMSD with different models and varying number of hidden layers. **(D)** Convergence trend of the training process using the variational autoencoder with 4 hidden layers.

Based on the number of layers in encoder and decoder modules, the number of neurons is adjusted to keep the same compression factor (ratio of sizes in adjacent layers) between layers. We refer to the model with *n* number of layers in the encoder as *n*-layer model (e.g., AE with 3 layers in the encoder as 3-layer AE). A total of 10 models (5 different layer numbers with 2 models) were trained using the training data and tested with the testing data. Each model was trained three times independently and the mean value of each metric was calculated. The results of performance assessment metrics are plotted in Figure 4.

The variational autoencoder with 4 hidden layers performed the best with high Spearman and Pearson coefficients and low RMSD.

In terms of the encoder (Figures 4A,B), a larger number of layers lead to a more complicated network that fail to keep enough biological information in the latent space. This is particularly evident in the autoencoders, in which both metrics drop sharply with increasing number of layers. In contrast, variational autoencoders kept a relatively flat curve. For the performance of the decoder (Figure 4C), variational autoencoders lead to a lower deviation between the training and decoded protein structures. Based on the elbow criteria, 4-layer VAE was selected as the final model with good performance and short training time. The convergence of the training process was evaluated using Spearman correlation coefficient, Pearson correlation coefficient, and RMSD as metrics with regard to the number of training iterations (epochs) used in the training process of 4-layer VAE. The convergence of these values is apparent when approaching 200 epochs (Figure 4D).

**FIGURE 5:**
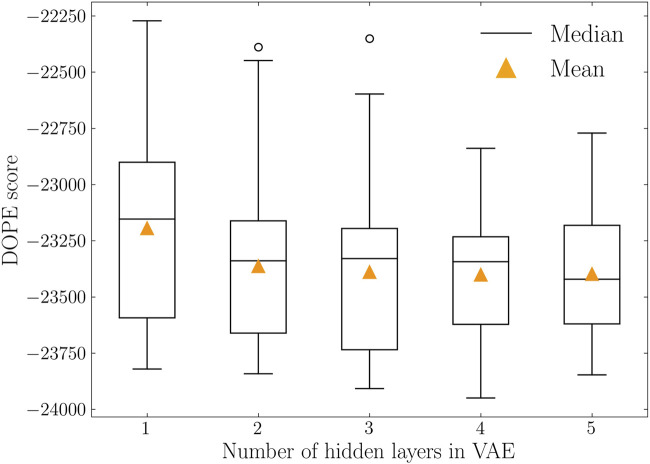
DOPE score distributions of variational autoencoders with varying number of hidden layers. Median and mean values are represented in solid black lines and orange triangles, respectively. 4-layer VAE reached the lowest DOPE score with mean value of −23,401.

To further evaluate the model performance in decoding protein conformations, two distance-based metrics were calculated and listed in Table 3. For both cases, the variational autoencoder with 4 hidden layers reached the lowest values. This indicates that 4-layer VAE is capable of generating protein conformations that are closer to the training distribution. In addition to the comparison of distributions, the quality of decoded protein conformations is quantified by DOPE score. The DOPE scores of decoded protein conformations were calculated with their distributions represented as boxplots in Figure 5. The mean DOPE score decreases from 1-layer VAE to 4-layer VAE, indicating the increased ability to generate protein conformations that have lower potential energy. This ability does not increase further with more complex model structure as shown by the 5-layer VAE.

**TABLE 3:** Variational autoencoder evaluation using MMD and EMD metrics. Variational autoencoder with *n* number of hidden layers is abbreviated as Vn. The lowest value in each metric is shown in bold font.

Metric	V1	V2	V3	V4	V5
MMD	0.0480	0.0171	0.0216	**0.0115**	0.0177
EMD	0.3221	0.2116	0.2225	**0.1476**	0.2144

To summarize, the above results suggest that a variational autoencoder with 4 hidden layers in both of the encoder and decoder modules exhibited the best performance in terms of learning a meaningful latent space and decoding physically plausible proteins conformations. Therefore, 4-layer VAE was chosen to conduct further analysis.

**FIGURE 6:**
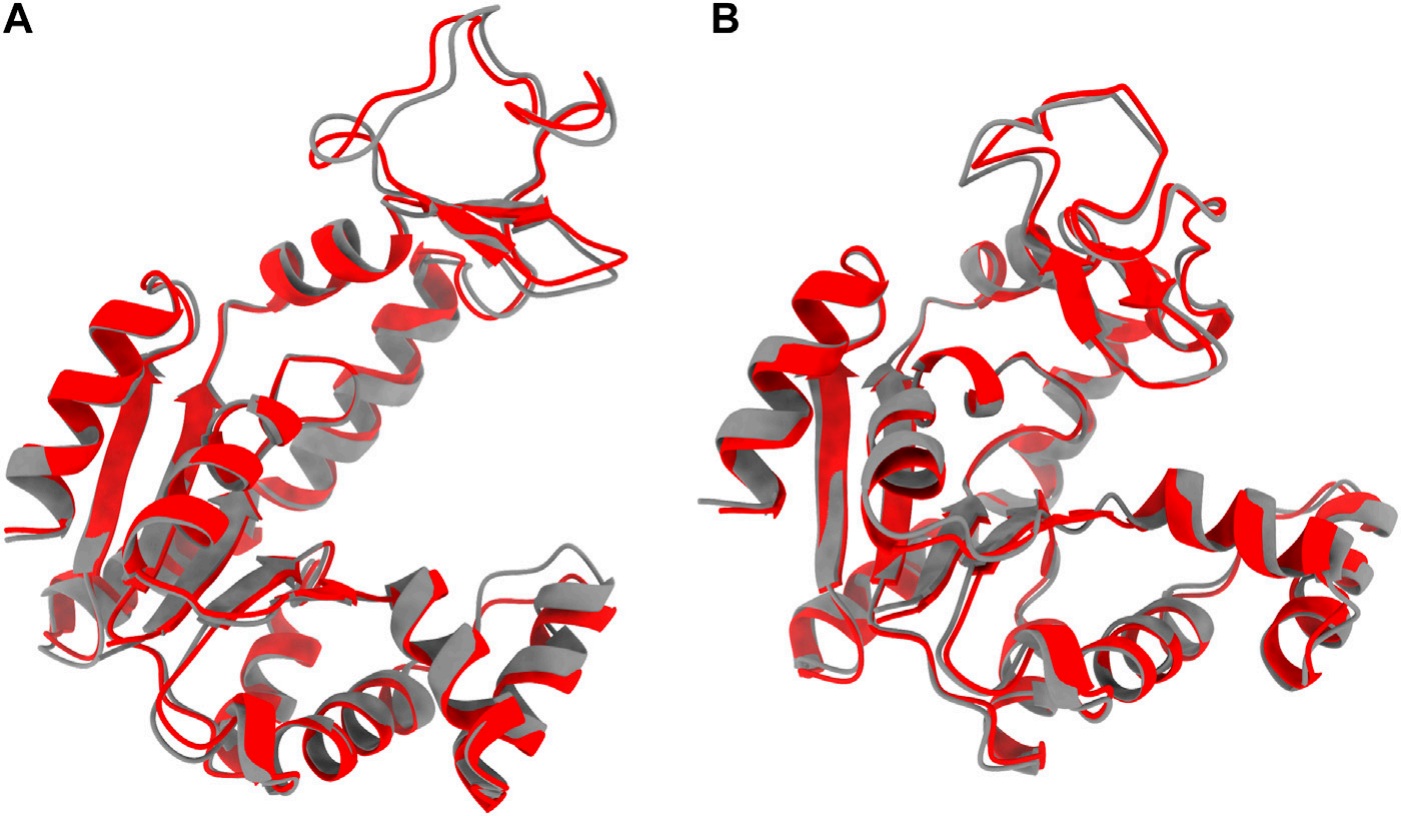
Comparison between the ADK native structures (grey) and decoded structures (red) in **(A)** the open state and **(B)** the closed state in the 4-layer VAE.

**FIGURE 7:**
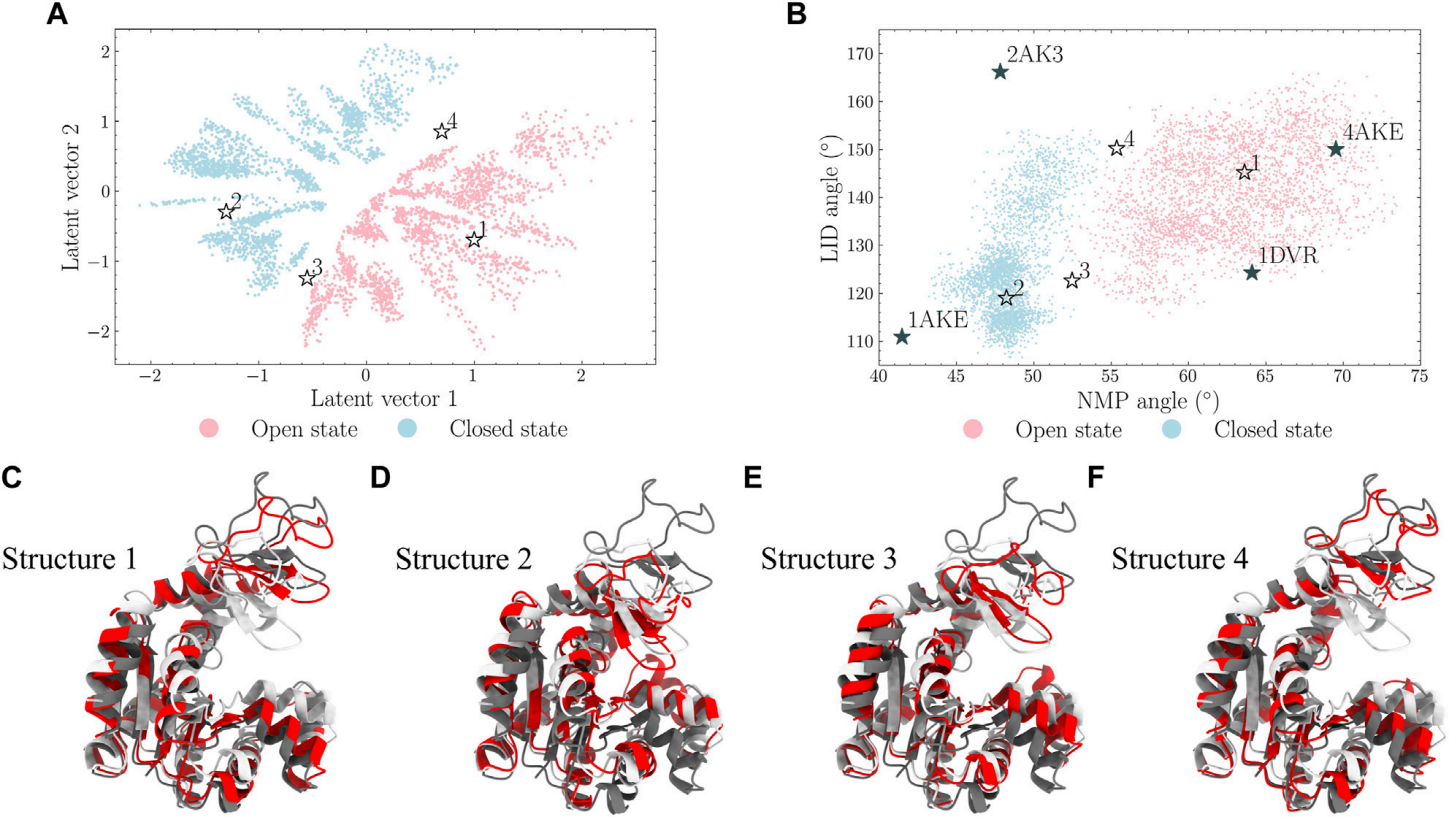
Four points [points 1-4 in **(A)** and **(B)**] in the latent space were selected and their decoded structures are displayed in **(C–F)**. It is shown that points 1 and 2 locate in the open and closed states, respectively. Points 3 and 4 in the frontiers of the latent space also locate in the intermediate regions of the protein angle map.

Two decoded ADK structures in the open and the closed states through the selected 4-layer VAE are illustrated in Figure 6. The mean RMSD between the training and decoded structures is 1.03 $\overset{{}^{\circ}}{A}$ . The learned latent space is plotted in Figure 7A. It is shown that the regions of the open and closed states are well separated. Also, there are blank spaces within each region. Four data points were manually selected and their decoded structures are illustrated in Figures 7C–F with the NMP-CORE and LID-CORE angles plotted in Figure 7B.

It should be noted that the latent space learned the nature of the characterizing angles as they shared similar trends. Points 1 and 2, originally selected from the open and closed states regions in the latent space, also lie in the regions of the open and closed states in the angle map, respectively; points 3 and 4, from the middle of two states in the latent space, also locate in the boundary of these two states in the angle map. This indicates that the learned latent space can be used to generate similar or different protein conformations by selecting nearby or distant points in the latent space, respectively.

To further explore the conformational spaces starting from the generated structures, additional 5 ns simulations, following the same procedure as described in the Molecular dynamics simulations section, were conducted using the decoded structures of points 3 and 4. For comparison, the training data set of 5 ns closed state simulations was extended to 50 ns.

**FIGURE 8:**
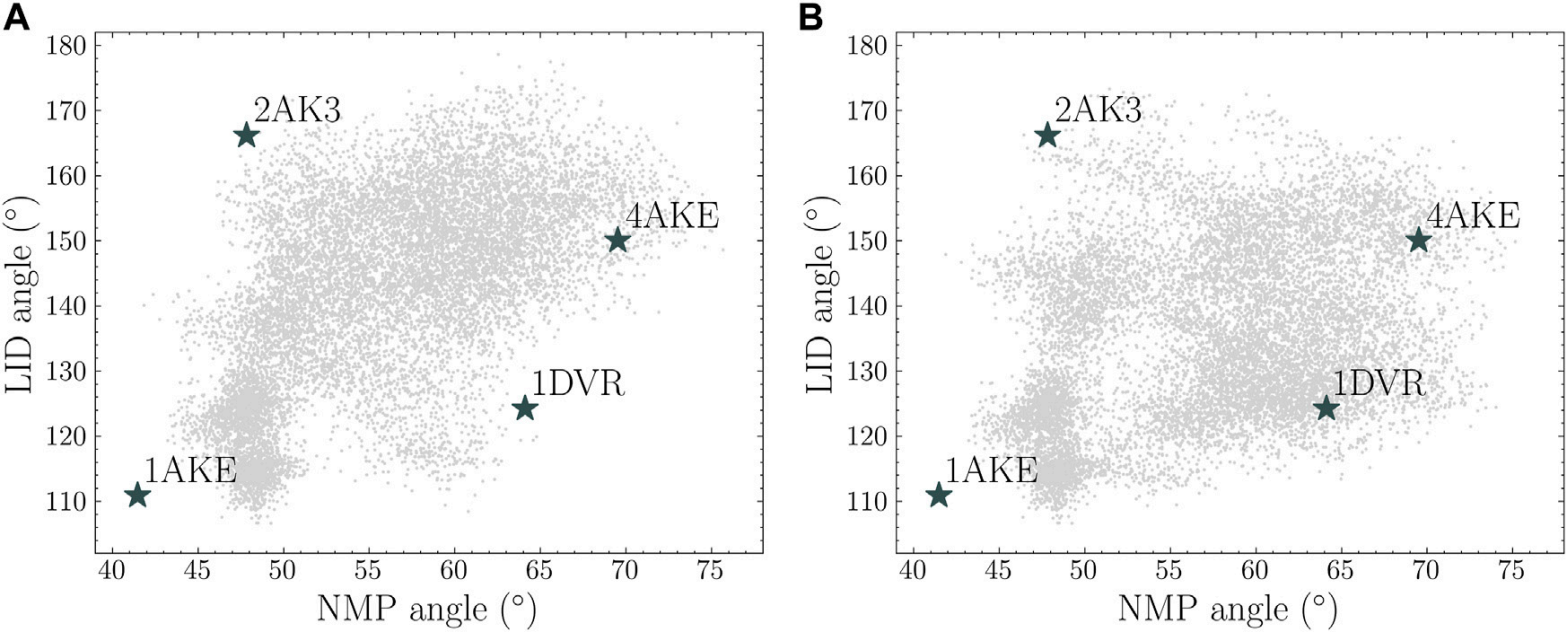
Protein conformational spaces from **(A)** 50 ns simulation from the closed state and **(B)** four sets of 5 ns simulation from the closed state, the open state and two generated conformations (point 3 and 4).

Two angle maps are plotted in Figure 8 to show the conformational spaces from the 50 ns closed state simulations and a combined MD simulations from the original 5 ns MD simulations in the open and closed states and the additional 5 ns simulations from the generated structures. It is shown that the MD simulations consisting of four short trajectories covered a similar conformational space compared with one long MD simulations. Both of these simulations explored the regions near the intermediate state of LID-open NMP-closed structure (PDB id: 2AK3). A full transition from the closed state to the open state can be constructed using both landscapes. Moreover, the combined simulations sampled hidden spaces near LID-closed NMP-open structure (PDB id: 1DVR) while these regions are less sampled in the long trajectory.

**FIGURE 9:**
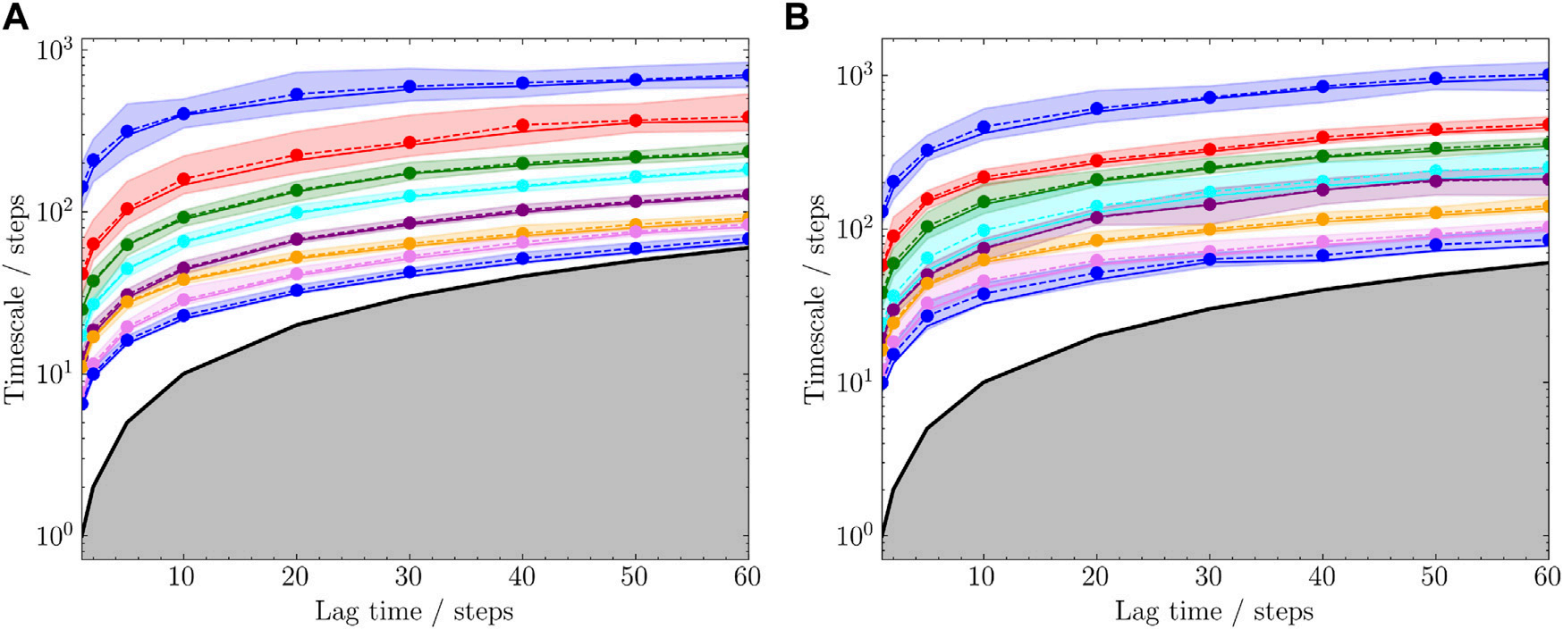
Estimated timescales with different lag times. The subplots **(A)** and **(B)** correspond to the trajectories in Figures 8A,B, respectively. Top 8 timescales were selected and each was plotted with 95% confidence interval.

To quantitatively compare the sampling efficiency, implied timescales in both trajectories were estimated based on the 2D coordinates on the angle maps. K-means clustering method was used with 100 cluster centers. Markov state models were built and the implied timescales were calculated for each trajectory. The results are shown in Figure 9. It is shown that the short combined simulations in Figure 8B exhibited similar implied timescales as the reference trajectory.

## 4 Discussion

The protein energy landscapes could be divided into many local energy minima which are represented as the metastable conformational states. These conformations are separated by free energy barriers that are much higher than *k*_*B*_ *T*. (He et al., 2003) Due to this reason, MD simulations are often trapped in a local minimum for a long simulation time before jumping to another. In this study, we aim to accelerate this inefficient process by directly taking protein structures from the less sampled regions as the initial structures for additional MD simulations. However, protein structures are high-dimensional data with the degrees of freedom as 3N in the Cartesian space. Unlike ADK protein which has known intrinsic collective variables (NMP-LID angles) to characterize protein conformational space, most proteins do not have such representations.

To overcome this problem, We proposed an application of variational autoencoders to sample protein conformational spaces. The model is demonstrated to capture the key variables in characterizing protein structures as the decoded conformations are similar to the training frames. This capability comes from the non-linear nature of variational autoencoders. As shown in the case of other methods (Das et al., 2006; Tribello et al., 2012), this leads to an improved ability to learn the movement of covalently bonded atoms (Degiacomi, 2019).

With the high accuracy in projecting low-dimensional data points back to high-dimensional protein structures, the latent space can be used to generate new and plausible protein structures not in the training space. Since the latent space holds a distance similarity—that is, the distances between points in the latent space are proportional to the deviations of their corresponding decoded protein structures—it can be used to produce either similar conformations by selecting points near the training set or distinct conformations from distant points. In the current study, both kinds of points were selected. The decoded protein structures from points near the training data are compared through visualization and LID NMP angle map. The produced protein structures from the intermediate regions could be used to start new MD simulations for additional sampling. This strategy led to highly efficient conformational spaces sampling with less computational cost. It should be noted that those data points were selected manually *via* latent space visualization in the current study. Automatic data selection for massive parallel simulations is possible within the framework of the current results.

We heuristically applied several metrics for quantification and comparison based on the previous studies. Specifically, the performance of encoder modules was determined by Spearman and Pearson correlation coefficients, as the encoder module can be treated as a dimensionality reduction technique and these two indicators have been widely used in such tasks. The performance of decoder modules was defined as the resemblance between the training and decoded protein conformations. RMSD and the DOPE score were used to quantify structure and system energy differences, respectively. The DOPE score has been used in the assessment of computationally generated models (Deka et al., 2015; Khare et al., 2019). A good model is expected to have a low DOPE score. We systematically compared the DOPE scores of generated protein conformations in different VAE settings. It is shown that a complicated model architecture with more hidden layers can generate protein conformations with lower DOPE scores, while this is converged after 4 hidden layers. We further compared the RMSD distributions between training and generated protein conformations with MMD and EMD indicators. Under both cases, 4-layer VAE achieved the lowest scores and was considered the best in representing protein conformational spaces.

Sampling efficiency is compared between the 20 and 50ns trajectories. Currently, this is defined as the implied timescale based on the sampled protein NMP-LID angles. It can be seen from Figure 8 that, compared with the complete reference trajectory, the 20ns trajectory sampled similar conformational regions within shorter time. Moreover, the implied timescale calculation reveals that we can observe markovian behavior from the 20ns sampling and get the “correct” timescales we would obtain from a 50ns simulation, showing that our assisted ML-based sampling strategy is able to capture biological-relevant transitions between conformational states with significant less sampling.

Through the angle plot (Figure 8) and the estimated timescale calculation (Figure 9), it is demonstrated that short MD simulations including trajectories starting from the generated conformations in the latent space could achieve the sampling efficiency comparable to a single long MD simulation. This suggests that iteratively conducting short MD simulations starting from conformations generated in the learned conformational space could serve as an alternative approach to extensive MD simulations.

## 5 Conclusion

In summary, we demonstrated the success of variational autoencoders in exploring protein conformational spaces through short molecular dynamics simulations. A well-trained variational autoencoder is capable of projecting trajectories onto a low-dimensional latent space, which can be used to produce realistic conformations, either similar or distant to the training frames, that are not in the training space. This capability allows the prediction of unsampled and physically plausible protein conformations. These conformations can be used as restarters for additional MD simulations to accelerate sampling process.

## Data Availability

The raw data supporting the conclusion of this article will be made available by the authors, without undue reservation.
